# Supplementary material for: Comparing diagnostic methods for historical arbovirus outbreaks: Insights from 19th century “dengue” epidemics
Source: PLoS Negl Trop Dis. 2025 Sep 26;19(9):e0013551. doi: 10.1371/journal.pntd.0013551 (PMC12468935; doi:10.1371/journal.pntd.0013551)
Supplement: Attachment — Submitted filename: Response to Reviewers v2.0.docx [file pntd.0013551.s002.docx]

We would like to thank the reviewers and editors for their thoughtful comments and constructive feedback, which have helped us to improve the clarity and rigor of our manuscript. Please find below our detailed responses to each point raised.

**Summary and General Comments**

**Reviewer #1:**

This manuscript by Bonifay and colleagues presenting an interesting historical analysis of past outbreaks of "dengue", for which the authors investigate evidence that they were actually caused by dengue virus, chikungunya virus, or possible other pathogens. This is a topic that frequently comes into discussion in the field, and hence the authors systematic approach to the question is a useful contribution to the field. Generally, they acknowledge and accept the various limitations of such an endeavour and do not overstate their findings or conclusions. Nonetheless, several revisions are needed:

1) Section 2.4: the authors state that "Each article was independently reviewed by one physician from each [adjudication] group, ensuring two readings per article. After reviewing the articles, the adjudicators categorized them". It is not clear from this if each article was categorized by the two physicians reviewing the article, or if the physicians presented their interpretation to the adjudication committee who then together determined the categorization, or something else. Additional information is needed to clarify this process. Of note, if only one physician classified each article for each committee, it calls into question the validity and utility of this part of the overall study.

Thank you for your comment. The articles have indeed been independently proofread by 2 different physicians. The sentence has been reworded to make it easier to understand.

*Each article was independently evaluated by two physicians, one from each committee, so that every article was reviewed twice by two distinct adjudicators. These physicians had unrestricted access to the full text of the articles but were blinded to each other’s assessments. Based on their independent review, each adjudicator categorized the article according to one of four predefined outcomes: 1. DENV infection; 2. CHIKV infection; 3. Other pathology; 4. Insufficient information to decide.*

2) The calculation of sensitivity, specificity, and accuracy should be removed from the manuscript, as there is no "gold standard" by which to compare any of the analytic components of this study. One could make a case for any of the three parts (scoring, readings, proofreading) to be compared to any other - I see no reason why the expert opinion proofreading should be seen as the most correct and used as the comparator. That said, I would appreciate if the authors were to present an analysis of which outbreaks were determined to be most likely caused by CHIKV or DENV via agreement of all three methods vs. those for which there were disagreement. (The authors present some such comparison already, but not agreement between all methods.)

Thank you for this thoughtful comment. We agree with your assessment: in the absence of a biological gold standard, none of the three methods (clinical score, expert readings, contextual proofreading) can be considered definitively correct. The contextual re-reading by specialists with historical and clinical expertise has been used in prior publications and seemed to represent the most widely accepted approach in this type of retrospective study. However, we fully acknowledge that it does not constitute a true gold standard.

In line with your suggestion, we have removed the calculations of sensitivity, specificity, and accuracy from the revised manuscript. Instead, we now emphasize the degree of agreement across methods. This addition, we hope, improves transparency and reinforces the exploratory nature of the analysis.

The Results and Discussion sections have been substantially revised in order to address your comments.

3) The format of the manuscript as a whole is atypical and (subject to the direction of the journal editors) may require significant re-formatting. Of note:

a) The use of numbered section headings (1, 2.3, 3.2.4, etc.) is not typical. Standard headings (Introduction, Materials and Methods, etc.) followed by non-numbered headings may be of greater utility to the reader.

b) The abstract does not follow the standard format of Intro/Methods/Results/Conclusions. I defer to the editors to determine if re-formatting is required or if the current format is acceptable.

c) Lines 230-232 belong in the Discussion.

d) Lines 251-255 belong in the Discussion.

e) Section 4 (Limitations) should be a component of the Discussion, not a free-standing section.

We thank the reviewer for their careful reading and helpful feedback regarding the manuscript’s structure. We agree that the format needed to align more closely with the journal’s standards. In response:

- We have removed the numbered section headings and adopted the conventional structure (Introduction, Methods, Results, Discussion).
- The content from lines 230–232 and 251–255 has been relocated to the Discussion section, where it is now more appropriately contextualized.
- The “Limitations” section has been fully integrated into the Discussion, rather than presented as a stand-alone section.

We hope these changes improved the manuscript's clarity and readability and align it better with the expectations of the journal and its readership.

**Reviewer #2:**

In this article, Bonifay et al attempt to assign the probably causal infectious aetiology of multiple historic “dengue” outbreaks. Using a series of literature reviews, scoring metrics, and input from current infectious disease clinicians, the authors posit that many historic “dengue” outbreaks that occurred before the identification of DENV may have been caused by other arboviruses, such as CHIKV.

My most significant concern with the methodology utilized in this study is that the clinical presentation of arboviral infections – such as DENV and CHIKV – can vary significantly over time and in different regions of the world, and additionally based on the exposure history of the population being examined. I have no doubt that the experts polled in this study are extremely competent in identifying the clinical symptoms of modern DENV and CHIKV infections, but it seems like a significant leap to assume that the clinical presentation of these infections would be identical 200+ years ago given wide variation in clinical presentation we see today. We know from modern serostudies that clinical diagnosis of even modern DENV infections can be very hit-or-miss without molecular, serologic, and/or antigenic diagnostics. It seems hard to believe that 2nd/3rd-hand diagnosis would be any more accurate.

We thank the reviewer for this important observation, which indeed highlights the major limitation of our study: the absence of biological confirmation for historical cases. However, we believe several elements support the relevance of our approach despite this inherent uncertainty:

- CHIKV and DENV were identified more than 75 and 80 years ago, respectively, and their clinical presentations have remained remarkably consistent over time. While our understanding of atypical or severe forms has improved, and the nuances of local epidemic dynamics are better documented, the core symptomatology and transmission patterns have shown little change.
- The temporal gap between the 19th century descriptions and the virological identification of CHIKV and DENV (1950s) is comparable to the time that has passed since their discovery. Yet, the clinical case definitions remain useful today, especially in epidemic settings. In routine clinical practice, molecular diagnostics are not always performed due to resource constraints and are typically reserved for patients at higher risk of complications.
- The historical descriptions we examined date primarily from the 19th century, and available phylogenetic data do not suggest major changes in CHIKV virulence or symptomatology since that period. While genetic drift and the emergence of different genotypic clusters—such as Asian and ECSA lineages—are well documented, no shift has been associated with a fundamentally altered clinical phenotype. In fact, it may be worthwhile in future work to further explore the possible relationship between specific genotypes and historical epidemics, such as the 1871–73 outbreak.
- Finally, to our knowledge, no other arbovirus has caused outbreaks of similar magnitude and geographic spread as DENV and CHIKV. New World arboviruses such as Mayaro virus (MAYV), while having epidemic potential, have remained geographically localized. Oropouche virus (OROV) appears capable of causing large-scale epidemics, sometimes affecting tens of thousands of individuals. However, it is associated with few reports of arthralgia. Clinically, it is difficult to distinguish from dengue virus infection, except perhaps for the characteristic biphasic symptom pattern occasionally described with Oropouche, which is not typically observed in dengue. Other Alphaviruses like O'nyong-nyong virus (ONNV) have caused large-scale epidemics in Africa but have shown limited geographic expansion. As for other Flaviviruses, such as Zika virus (ZIKV), although capable of widespread transmission, their clinical presentations are generally milder and often go unnoticed, reducing the likelihood of being mistaken for the more symptomatic "dengue-like" syndromes described historically.

Of course, while the second half of the 19th century is relatively recent, we are aware that other suspected CHIKV epidemics occurred earlier, which we did not address in this study. For example, an epidemic was reported in 1780 on the Indian subcontinent. However, the available descriptions are limited and lack detail. Finally, we do not claim to bring certainties, but rather to offer a reflection on how we interpret historical epidemics—especially at a time when, despite technological advances and accelerating globalization, epidemic dynamics have shown remarkable similarities over the past 200 years.

**Reviewer #3:**

The authors have compiled a reasonably complete bibliography of the nearly 250-year-old literature describing outbreaks of short duration febrile diseases caused by chikungunya and dengue viruses. It is not merely confusing, but somewhat short-shrifting for the authors to fail to preface this review by acknowledging dengue-expert Donald Carey’s 1970 trip to the Harvard Library where he had recognized that the 1779 “dengue” epidemic in Batavia, New Netherlands closely resembled chikungunya febrile disease. In the medical literature, it had long been labeled an outbreak of dengue. From this insight, he wrote “Chikungunya and “dengue,” a case of mistaken identity?” published in 1971. Equally important, a report by another arbovirologist, Scott Halstead, published in 2015, recognized that chikungunya in the Caribbean was a reappearance of a chikungunya virus that had been transported from East Africa in 1827 to the Caribbean where the disease it caused had been given the name, “dengue.” This article, rich in historical details, was published in 2015.

We are honored and grateful to Dr. Halstead for reviewing our manuscript.

We sincerely acknowledge the foundational role that his 2015 article played in shaping our thinking about the 1827–28 epidemic in the Caribbean. We also fully recognize the pioneering work of Donald Carey, as presented in his 1971 article. Both are already cited in our manuscript: Carey’s hypothesis regarding the misidentification of chikungunya as “dengue,” and Dr. Halstead’s proposal that the 1827 Caribbean outbreak may have been caused by CHIKV introduced from East Africa.

That said, we understand that the introductory section did not sufficiently highlight the central importance of these historical insights. In response, we have revised the introduction to better foreground these contributions and to acknowledge more explicitly how our study builds upon this existing scholarship. We appreciate this opportunity to strengthen our historical framing and to clarify the continuity between previous work and our own.
