# Supplementary material for: Comparing diagnostic methods for historical arbovirus outbreaks: Insights from 19th century “dengue” epidemics
Source: PLoS Negl Trop Dis. 2025 Sep 26;19(9):e0013551. doi: 10.1371/journal.pntd.0013551 (PMC12468935; doi:10.1371/journal.pntd.0013551)
Supplement: Attachment — Submitted filename: Response to Reviewers.docx [file pntd.0013551.s003.docx]

**Response to the reviewer**

Reviewer #3: The reviewer offers as a “gift” sentences from his own history of chikungunya. “The names, “Dengue” and “dengue fever” were attached to distinct short-duration febrile diseases before their viral etiology was established. The clinical term “dengue” entered the medical literature in the American hemisphere in the 19th century describing a short-duration febrile exanthem. It was not an English word, but a Spanish homonym of the African Swahili “kidinga pepo,” sometimes pronounced “denga” or “dyenga” This was the name given to epidemics of a disabling febrile arthritis, now known as chikungunya, during outbreaks in East Africa in 1823 and again in 1870. (1) In 1881, James Christie, a Scottish-trained physician attached to the Sultan of Zanzibar, in the 1860s-70s published a report explicitly linking an 1823 East African outbreak of kidinga pepo to the outbreak in 1827 – 28 of “dengue” in the Americas. (2) In commenting on the dispersal of what we now know to be chikungunya virus, Christie described reports that the 1823 Zanzibar epidemic had spread East to Gujarat, India, then on to Calcutta and in 1824 to Rangoon. He added that there had been a similar spread of the 1823 “dengue” epidemic, this time westwards. In 1827, this epidemic reached St.Thomas in the West Indies. Christie states “I am of the opinion that both the disease and its designation were imported in the West Indian Islands direct from the East Coast of Africa.” (2) He noted it was then that the medical term “dengue” was introduced into the West Indies as a Spanish homynym of the African word. Another observer, contemporary to the West Indian epidemic, agreed. He wrote: “when the dengue” reached New Orleans in 1828, “the disease alluded to is supposed to have been brought from Africa, with some slaves imported into the Havana. In that place it obtained the name of Dingee, Dengue, Danga, etc. It was there very prevalent and also in Barbadoes, where it received the name of Dandy fever, from the stiffened form and dread of motion in patients.” (3)
1. Christie J. Remarks on "kidinga Pepo"' a peculiar form of exantematous disease. Epidemic in Zanzibar, East Coast of Africa, from July 1870 till January 1871. BMJ. 1872;1:577-79.
2. Christie J. On epidemics of dengue fever: their diffusion and etiology. Glasgow Med J. 1881;3:161-76.
3. Dumaresq PJ. An account of dengue, danga or dandy fever, as it occurred in New Orleans and in the person of the writer,communicated in a letter to one of the editors. . Boston Medical and Surgical Journal. 1828;1:497-502.
The reviewer hopes the authors will take the time to investigate the outbreak of 1900 in Singapore which extended throughout southern Asia. This has been confused over and over as caused by a dengue virus. Clearly, it is chikungunya. Remember, chikungunya is an African zoonosis that spills over into an urban cycle on the East coast of Africa at approximately 40 year intervals. It has made the transit across the Indian Ocean many, many times.

Thank you for your comments and for the contribution. The paragraph has been added to the manuscript. Regarding the 1900 epidemic in Singapore, this example has been added to the discussion section as an opening, highlighting that the different methods used in our study could be applied to other epidemics with still-debated etiologies. We fully agree with the reviewer on the richness of 19th-century medical literature and the importance of continuing to bring it to light.

**Note to the editor:** *The bibliography has been reorganized, but no new references have been added. The articles mentioned by the reviewer were already included in the original reference list.*
